# Fatty Acid Amide Hydrolase-Dependent Generation of Antinociceptive Drug Metabolites Acting on TRPV1 in the Brain

**DOI:** 10.1371/journal.pone.0070690

**Published:** 2013-08-05

**Authors:** David A. Barrière, Christophe Mallet, Anders Blomgren, Charlotte Simonsen, Laurence Daulhac, Frédéric Libert, Eric Chapuy, Monique Etienne, Edward D. Högestätt, Peter M. Zygmunt, Alain Eschalier

**Affiliations:** 1 Clermont Université, Université d’Auvergne, Pharmacologie Fondamentale et Clinique de la Douleur, Laboratoire de Pharmacologie, Facultés de Médecine/Pharmacie, Clermont-Ferrand, France; 2 Inserm, U1107 Neuro-Dol, Clermont-Ferrand, France; 3 Department of Clinical Chemistry and Pharmacology, Lund University, Lund, Sweden; 4 CHU Clermont-Ferrand, Service de Pharmacology, Hôpital G. Montpied, Clermont-Ferrand, France; Sapienza University of Rome, Italy

## Abstract

The discovery that paracetamol is metabolized to the potent TRPV1 activator N-(4-hydroxyphenyl)-5Z,8Z,11Z,14Z-eicosatetraenamide (AM404) and that this metabolite contributes to paracetamol’s antinociceptive effect in rodents via activation of TRPV1 in the central nervous system (CNS) has provided a potential strategy for developing novel analgesics. Here we validated this strategy by examining the metabolism and antinociceptive activity of the de-acetylated paracetamol metabolite 4-aminophenol and 4-hydroxy-3-methoxybenzylamine (HMBA), both of which may undergo a fatty acid amide hydrolase (FAAH)-dependent biotransformation to potent TRPV1 activators in the brain. Systemic administration of 4-aminophenol and HMBA led to a dose-dependent formation of AM404 plus N-(4-hydroxyphenyl)-9Z-octadecenamide (HPODA) and arvanil plus olvanil in the mouse brain, respectively. The order of potency of these lipid metabolites as TRPV1 activators was arvanil = olvanil>>AM404> HPODA. Both 4-aminophenol and HMBA displayed antinociceptive activity in various rodent pain tests. The formation of AM404, arvanil and olvanil, but not HPODA, and the antinociceptive effects of 4-aminophenol and HMBA were substantially reduced or disappeared in FAAH null mice. The activity of 4-aminophenol in the mouse formalin, von Frey and tail immersion tests was also lost in TRPV1 null mice. Intracerebroventricular injection of the TRPV1 blocker capsazepine eliminated the antinociceptive effects of 4-aminophenol and HMBA in the mouse formalin test. In the rat, pharmacological inhibition of FAAH, TRPV1, cannabinoid CB1 receptors and spinal 5-HT_3_ or 5-HT_1A_ receptors, and chemical deletion of bulbospinal serotonergic pathways prevented the antinociceptive action of 4-aminophenol. Thus, the pharmacological profile of 4-aminophenol was identical to that previously reported for paracetamol, supporting our suggestion that this drug metabolite contributes to paracetamol’s analgesic activity via activation of bulbospinal pathways. Our findings demonstrate that it is possible to construct novel antinociceptive drugs based on fatty acid conjugation as a metabolic pathway for the generation of TRPV1 modulators in the CNS.

## Introduction

Paracetamol is an effective analgesic, a single oral dose of 1000 mg having an NNT (numbers needed to treat) of 3.6 for at least 50% pain reduction over 4–6 hours in patients with postoperative pain [1]. However, the formation of toxic metabolites, such as N-acetyl-*p*-benzoquinone imine (NAPQI), in the liver is becoming an increasing concern particularly during long-term use, potentially preventing the full exploitation of paracetamol’s analgesic activity. We have previously demonstrated that paracetamol is metabolized to the TRPV1 activator AM404 and that activation of TRPV1 in the brain by this metabolite contributes to the antinociceptive activity of oral paracetamol in rodents [2]–[4]. Mapping the *in vivo* metabolism of paracetamol in different organs indicated an extensive de-acetylation to the primary amine 4-aminophenol in the liver [2]. This well-known paracetamol metabolite enters the brain, where it undergoes conjugation with arachidonic acid to yield AM404, a reaction dependent on the enzyme FAAH [2]. The expression pattern in adult animals and the close evolutionary development of TRPV1 and FAAH implicate a functional relationship between these proteins in the nociceptive pathways [5]–[8]. Thus, the design of molecules undergoing FAAH-dependent biotransformation to TRPV1 active drug metabolites in the CNS represents a plausible strategy for developing novel antinociceptive agents that may be more effective and less toxic than paracetamol.

Here we validated this strategy by examining whether systemic administration of the primary amines 4-aminophenol, the de-acetylated metabolite of paracetamol, and HMBA, none of which are directly oxidized to NAPQI, can produce antinociception via the generation of TRPV1 active drug metabolites in the brain. Using mass spectrometry and various rodent nociceptive tests, we show that both 4-aminophenol and HMBA produce antinociception and undergo fatty acid conjugation in the brain, leading to the formation of AM404 plus HPODA and arvanil plus olvanil, respectively, of which arvanil and olvanil are extremely powerful TRPV1 activators with potencies in the subnanomolar range [9]–[11]. As shown for paracetamol, the antinociceptive effects of 4-aminophenol were dependent on FAAH, TRPV1, cannabinoid CB1 receptors and serotonergic mechanisms [3], [12], supporting our previous suggestion that 4-aminophenol is a key intermediate metabolite in the bioactivation of paracetamol.

## Results

### FAAH-dependent Metabolism of 4-aminophenol and HMBA

**Figure 1:**
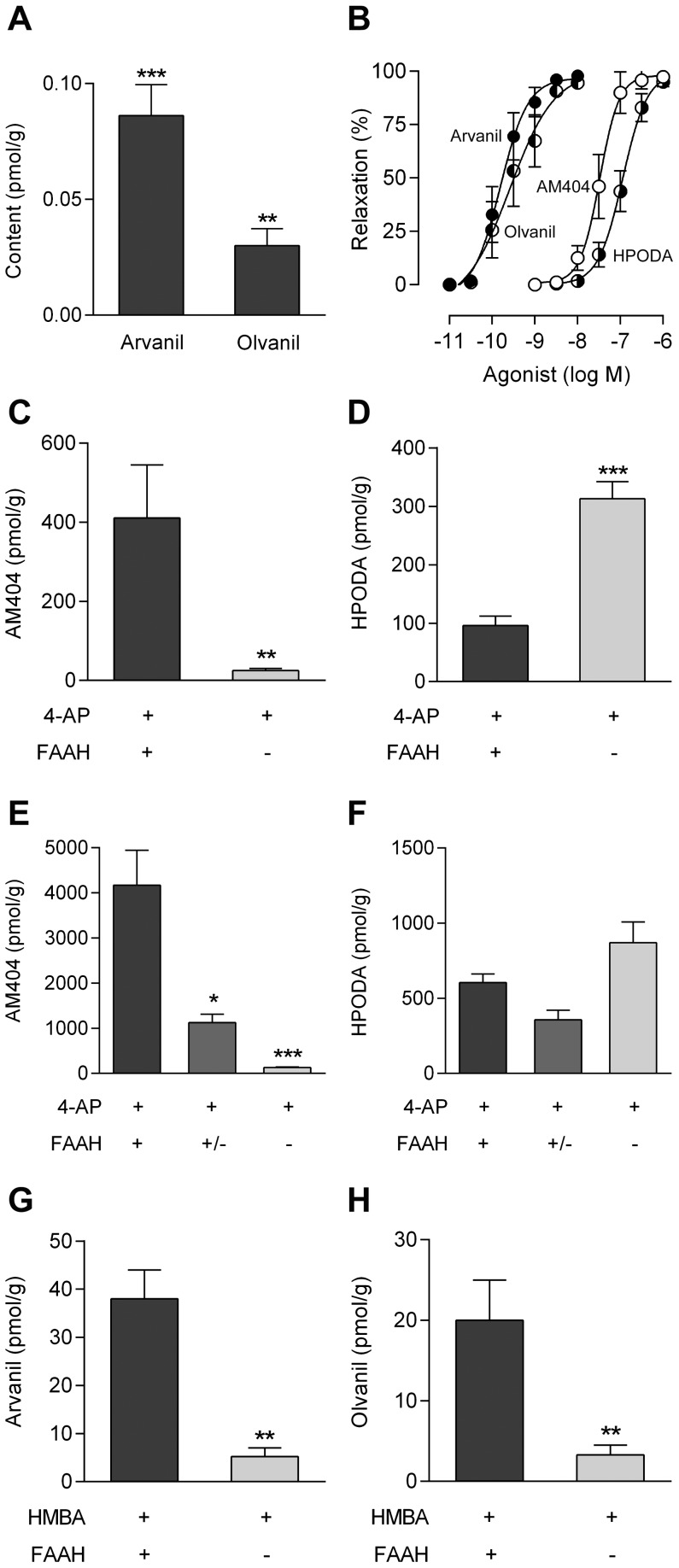
Production of TRPV1 active drug metabolites in the mouse brain from 4-aminophenol and 4-hydroxy-3-methoxybenzylamine (HMBA). Formation of arvanil and olvanil in rat brain homogenates incubated with HMBA (100 µM) for 60 min (**A**; n = 6). These N-acylamines were not detected in brain homogenates exposed to vehicle only. TRPV1-dependent vasorelaxation evoked by AM404 (n = 6), N-(4-hydroxyphenyl)-9Z-octadecenamide (HPODA; n = 9), arvanil (n = 12) and olvanil (n = 7) in rat mesenteric arterial segments precontracted with phenylephrine (**B**). Content of AM404 (**C** and **E**) and HPODA (**D** and **F**) in the brain 20 min after intraperitoneal injection of 4-aminophenol at doses of 30 mg/kg (**C** and **D**; n = 5) and 100 mg/kg (**E**; n = 6–12 and **F**; n = 6) in different FAAH genotypes. Contents of arvanil (**G**; n = 6) and olvanil (**H**; n = 6) in the brain 20 min after intraperitoneal injection of HMBA at a dose of 300 mg/kg in different FAAH genotypes. Values are expressed as mean ± SE. *p<0.05, **p<0.01 and ***p<0.001 when compared to wild-type littermates, using Mann-Whitney *U* test (**A**, **C**, **D**, **G** and **H**) or Kruskal-Wallis one-way ANOVA followed by Dunn’s multiple comparisons test (**E** and **F**).

The primary amine 4-aminophenol is an excellent substrate for FAAH in the biosynthesis of AM404 in brain homogenates from mice and rats [2]. Here we show that incubation of mouse brain homogenates with another primary amine, HMBA, leads to the formation of arvanil and olvanil (Fig. 1A), which are both recognized as potent TRPV1 activators [9]–[11]. To establish whether fatty acid conjugation of 4-aminophenol and HMBA also occurs in living animals, we determined the contents of AM404, HPODA, arvanil and olvanil in the brain 20 min after intraperitoneal injection of 4-aminophenol (30 and 100 mg/kg) and HMBA (100 and 300 mg/kg) in mice. These N-acylamines were all found to be potent TRPV1 activators (Fig. 1B), arvanil (pEC_50_ = 9.7±0.1, n = 12) and olvanil (pEC_50_ = 9.5±0.2, n = 7) being 172 and 110 times more potent than AM404 (pEC_50_ = 7.5±0.1, n = 6), respectively, whereas AM404 was 3.3 times more potent than HPODA (pEC_50_ = 7.0±0.1, n = 9), using capsazepine-sensitive vasorelaxation as readout (Fig. S1). AM404 and HPODA as well as arvanil and olvanil were detected after administration of both doses of 4-aminophenol and HMBA, respectively (Table 1 and 2), but not after vehicle injection (n = 6). However, olvanil only surpassed the level of quantification after the high dose of HMBA (300 mg/kg).

**Table 1:** Quantification of 4-AP, AM404 and HPODA 20 min after 4-AP injection (i.p.).

	Blood	Brain		
4-AP (dose)#	4-AP (nmol/g)	4-AP (nmol/g)	AM404 (pmol/g)	HPODA (pmol/g)
30 mg/kg	283±15	91±12	411±134	96±16
100 mg/kg	878±157	7470±1720	4829±1435	797±114

4-AP, 4-aminophenol; HPODA, N-(4-hydroxyphenyl)-9Z-octadecenamide; i.p., intraperitoneal.

# n = 5-6 mice.

**Table 2:** Quantification of HMBA, arvanil and olvanil 20 min after HMBA injection (i.p.).

	Blood	Brain		
HMBA (dose)#	HMBA (nmol/g)	HMBA (nmol/g)	Arvanil (pmol/g)	Olvanil (pmol/g)
100 mg/kg	220±54	132±73	3.33±0.33	<LoQ*
300 mg/kg	2206±67	3895±284	56±10	45±5.6

HMBA, 4-hydroxy-3-methoxybenzylamine; i.p., intraperitoneal.

# n = 6 mice.

* Below the level of quantification (LoQ).

We also determined the contents of 4-aminophenol and HMBA in blood and the brain from the same animals (Table 1 and 2). A conspicuous difference between 4-aminophenol and HMBA was observed in the quotient between the brain levels of these molecules and their lipid metabolites, a parameter most likely reflecting the efficiency of the conjugation reaction. The quotients between 4-aminophenol and AM404 (HPODA) were 223 (951) and 1,547 (9,376) for the 30 mg/kg and 100 mg/kg doses of 4-aminophenol, respectively. The corresponding quotients between HMBA and arvanil (olvanil) were 39,773 (not determined) and 69,980 (86,307) for the 100 mg/kg and 300 mg/kg doses of HMBA, respectively. At an identical dose of 4-aminophenol and HMBA (100 mg/kg), the brain content of AM404 was approximately three orders of magnitude higher than that of arvanil (Table 1 and 2). Furthermore, the quotient between the contents of AM404 and 4-aminophenol (1∶1,500) was substantially larger than the quotient between the contents of arvanil and HMBA (1∶40,000), again indicating a more efficient conversion of 4-aminophenol to AM404 than of HMBA to arvanil in the mouse brain.

We have previously demonstrated that the formation of AM404 in the mouse brain is dependent on FAAH after systemic administration of paracetamol [2]. Here we show that the brain contents of AM404, arvanil and olvanil, but not HPODA, were substantially reduced in FAAH*^−/−^* mice compared to their FAAH*^+/+^* littermates 20 min after intraperitoneal injection of 4-aminophenol (30 and 100 mg/kg; Fig. 1C and E) and HMBA (300 mg/kg; Fig. 1G and H). We could not extend these findings to the low dose of HMBA (100 mg/kg), because the brain contents of olvanil and arvanil were either below or immediately above the level of quantification in wild-type mice, respectively. Notably, the brain content of HPODA increased 3-fold in FAAH*^−/−^* mice compared to their wild-type littermates after the low dose of 4-aminophenol, while no significant difference was found between the genotypes after the high dose of 4-aminophenol (Fig. 1D and F).

**Figure 2:**
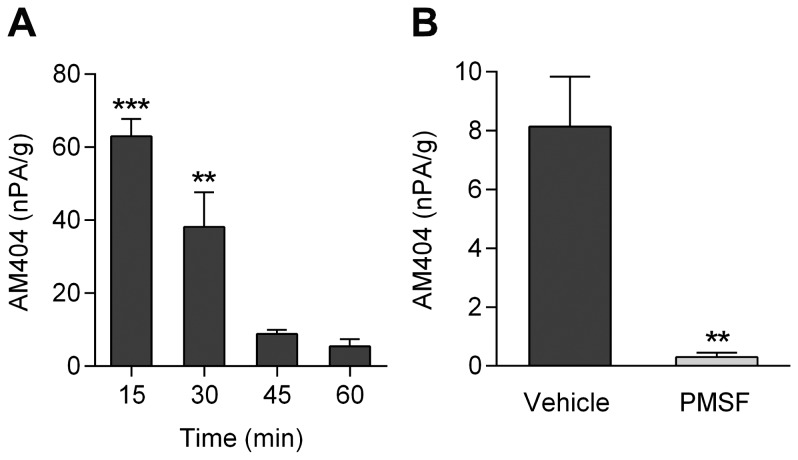
Content of AM404 in the rat brain after intraperitoneal injection of 4-aminophenol (100 mg/kg). Brain content of AM404 at different time points after 4-aminophenol administration (**A**; n = 6). Brain content of AM404 20 min after 4-aminophenol injection in animals pretreated with phenylmethanesulfonyl fluoride (PMSF; 10 mg/kg s.c.) 20 min before 4-aminophenol administration (**B**; n = 6). The AM404 content is presented as normalized peak area (nPA) per g protein. Results are expressed as mean ± SE. **p<0.01 and ***p<0.001 when compared to time zero (**A**) or vehicle-treated animals (**B**), using Kruskal-Wallis one-way ANOVA followed by Dunn’s multiple comparisons test (**A**) or Mann-Whitney *U* test (**B**).

The content of AM404 in brain was also determined at different time points after intraperitoneal administration of 4-aminophenol (100 mg/kg), as shown in the rat. Significant amounts of AM404 were detected 15 min and 30 min after 4-aminophenol injection (Fig. 2A). Pretreatment of the animals with the FAAH inhibitor phenylmethanesulfonyl fluoride (PMSF; 10 mg/kg s.c.) substantially reduced the formation of AM404, as measured 20 min after 4-aminophenol administration (Fig. 2B). These findings show that the *in vivo* condensation of 4-aminophenol and arachidonic acid is dependent on FAAH and support our previous notion that 4-aminophenol is a key intermediate metabolite in the bioactivation of paracetamol in the rodent brain [2], [3], [12].

### FAAH- and TRPV1-dependent Antinociceptive Effects of 4-aminophenol and HMBA

**Figure 3:**
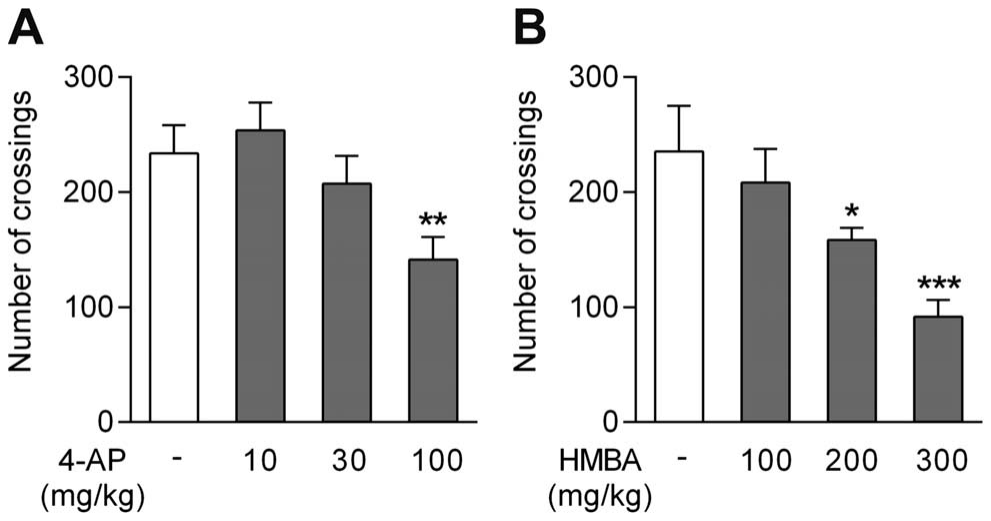
Effects of 4-aminophenol and 4-hydroxy-3-methoxybenzylamine (HMBA) on spontaneous locomotor activity in the mouse. Locomotor activity was assayed by counting the number of crossings of light beams in actimetry boxes over a 15 min period. Doses of 4-aminophenol (**A**; n = 4) and HMBA (**B**; n = 6) below 100 mg/kg and 200 m/kg, respectively, failed to impair locomotor activity. 4-Aminophenol and HMBA were injected by the intraperitoneal route 10 min before the test. *p<0.05, **p<0.01 and ***p<0.001 when compared to vehicle injection, using Kruskal-Wallis one-way ANOVA followed by Dunn’s multiple comparisons test.

We first tested the effects of 4-aminophenol and HMBA on locomotor activity, a potential bias in the assessment of antinociceptive activity. Locomotor activity was assessed 10 min after an intraperitoneal injection of 4-aminophenol (10, 30 and 100 mg/kg in mice) and HMBA (100, 200 and 300 mg/kg in mice) or vehicle. Mice showed reduced locomotor activity after the highest dose of 4-aminophenol (Fig. 3A), whereas rats tolerated all three doses (data not shown). For HMBA, only the dose of 100 mg/kg failed to affect locomotion (Fig. 3B).

#### Involvement of FAAH

**Figure 4:**
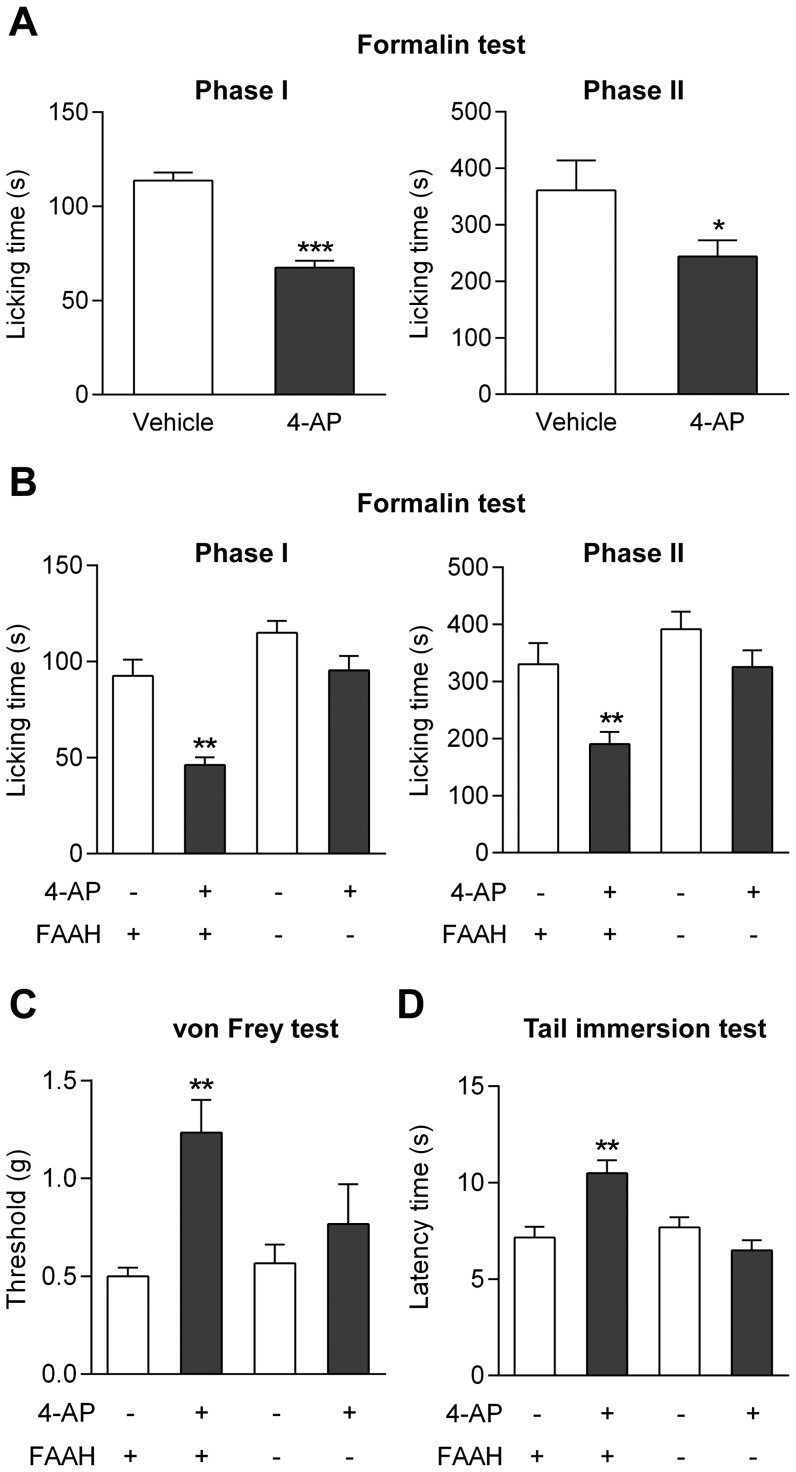
The antinociceptive effect of 4-aminophenol is dependent on fatty acid amide hydrolase (FAAH) in mouse. The antinociceptive effect of 4-aminophenol, given at a dose that did not affect spontaneous locomotor activity (Fig. 3), was lost in FAAH^−/−^ mice as compared to their wild-type littermates. The effect of 4-aminophenol at a dose of 30 mg/kg (i.p.) was assessed in the formalin test in C56BL/6 wild-type mice (**A**; n = 8) and in the formalin (**B**; n = 6–9), von Frey (**C**; n = 6) and tail immersion (**D**; n = 6) tests in FAAH^−/−^ mice and their wild-type littermates. 4-Aminophenol was injected by the intraperitoneal route 10 min before the tests. *p<0.05, **p<0.01 and ***p<0.001 when compared to vehicle injection, using Mann-Whitney *U* test.

As demonstrated in FAAH^+/+^ mice, 4-aminophenol at a dose of 30 mg/kg (i.p.) reduced the licking time during the first and second phases of the formalin test, increased the withdrawal threshold in the von Frey test and the latency time in the tail immersion test, indicating a clear antinociceptive activity (Fig. 4A–D). If 4-aminophenol is a key intermediate in the bioactivation of paracetamol, the antinociceptive effect of 4-aminophenol should be dependent on FAAH. Indeed, this was found to be the case, as 4-aminophenol failed to produce antinociception in all three pain tests in FAAH^−/−^ mice (Fig. 4B–D).

**Figure 5:**
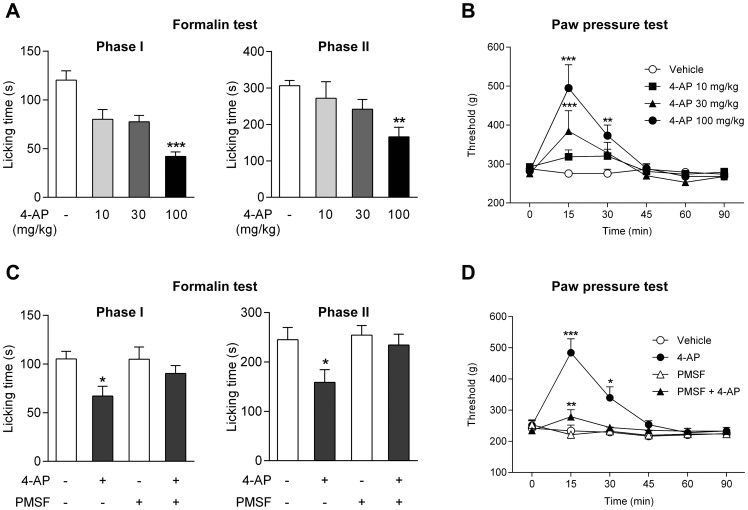
The antinociceptive effect of 4-aminophenol is dependent on fatty acid amide hydrolase (FAAH) in rats. 4-Aminophenol at a dose of 100 mg/kg significantly reduced the first and second phases of the formalin test (**A**; n = 7–8). Both the 30 mg/kg and the 100 mg/kg doses of 4-aminophenol increased the withdrawal threshold in the paw pressure test (**B**; n = 8). Pre-treatment with phenylmethanesulfonyl fluoride **(**PMSF; 10 mg/kg) substantially reduced or prevented the antinociceptive effect of 4-aminophenol (100 mg/kg) in the formalin (**C**; n = 10–14) and paw pressure (**D**; n = 7–11) tests. The different doses of 4-aminophenol were injected by the intraperitoneal route 10 min before the tests. PMSF was injected subcutaneously 20 min before 4-aminophenol administration. *p<0.05, **p<0.01 and ***p<0.001 when compared to vehicle injection, using Kruskal-Wallis one-way ANOVA followed by Dunn’s test (**A**), repeated measures two-way ANOVA followed by Dunnetts (**B**) or Sidak’s (**D**) multiple comparisons tests, or Mann-Whitney *U* test (**C**).

In the rat, the effects of three doses of 4-aminophenol (10, 30 and 100 mg/kg i.p.) were examined in the formalin test. Only the highest dose reduced both phases of the formalin test, as measured by the licking time (Fig. 5A). This dose of 4-aminophenol also increased the withdrawal threshold in the rat paw pressure test 15 and 30 min after drug administration, while the intermediate dose (30 mg/kg) was effective only at 15 min and the low dose (10 mg/kg) was inactive in this test of acute mechanical nociception (Fig. 5B).

We have previously reported that pretreatment with the FAAH inhibitor PMSF (10 mg/kg s.c.) prevents the antinociceptive activity of paracetamol in the rat [12]. Here we show that PMSF also inhibited the antinociceptive effect of 4-aminophenol (100 mg/kg i.p.) in the rat formalin and paw pressure tests (Fig. 5C and D). Although PMSF may inhibit several serine proteases, these findings are consistent with 4-aminophenol being a key intermediate metabolite contributing to the antinociceptive action of paracetamol.

**Figure 6:**
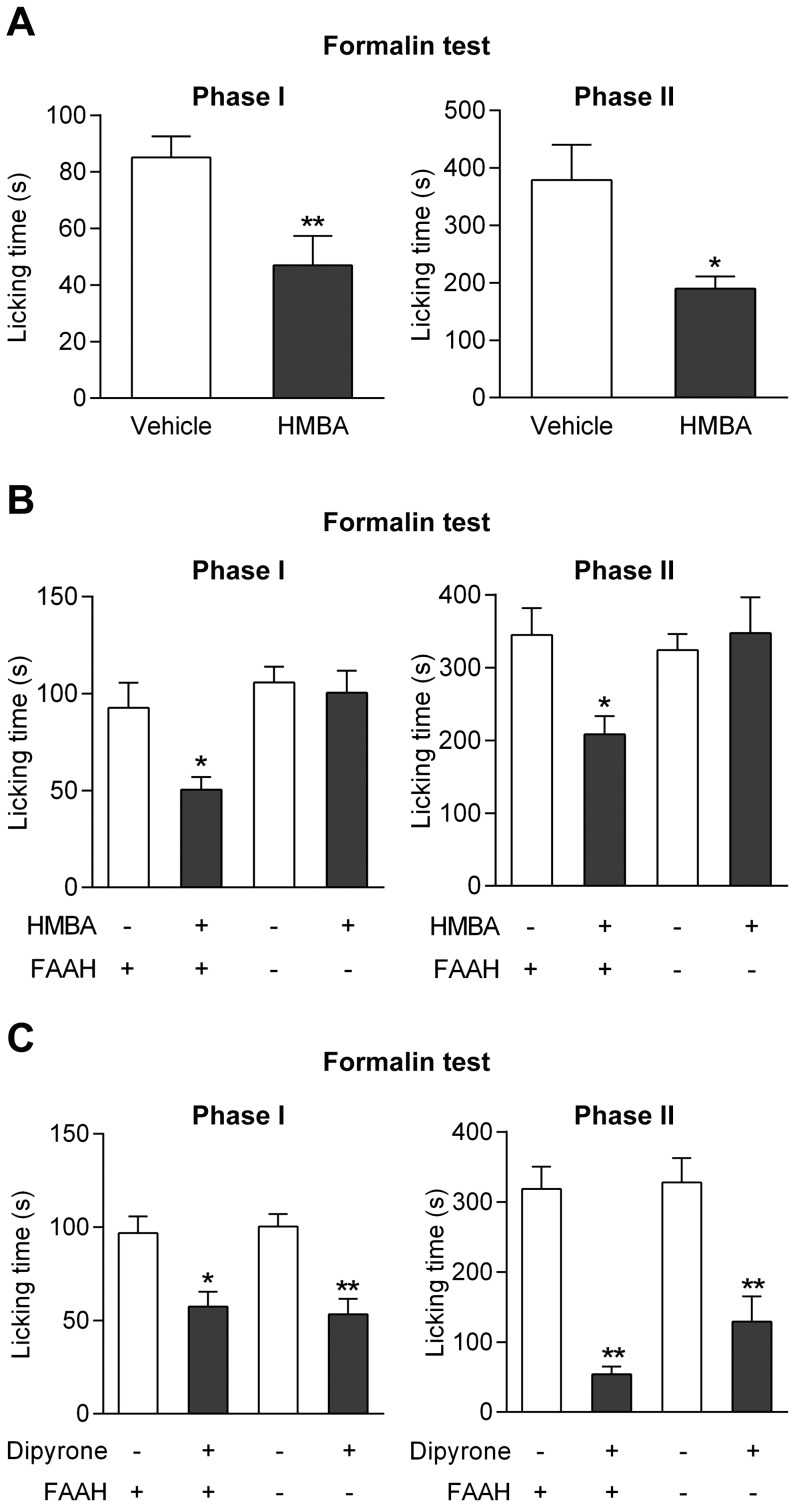
Effects of 4-hydroxy-3-methoxybenzylamine (HMBA) on nociception in the formalin test in mice. At a dose that did not affect spontaneous locomotion (Fig. 3B), HMBA (100 mg/kg) inhibited nocifensive behaviour in both phases of the formalin test in C57BL/6 wild-type mice (**A**; n = 5–7). The antinociceptive effect of HMBA disappeared in FAAH^−/−^ mice as compared to their wild-type littermates (**B**; n = 4–8), whereas dipyrone (30 mg/kg) produced similar antinociception in FAAH^+/+^ and FAAH^−/−^ mice (**C**; n = 5–7). HMBA and dipyrone were injected by the intraperitoneal route 10 min before the tests. *p<0.05 and **p<0.01 when compared to vehicle injection, using Mann-Whitney *U* test.

We further examined the effect of the primary amine HMBA in the mouse formalin test. As this drug is metabolized to the ultrapotent TRPV1 activators arvanil and olvanil in the rodent brain, we expected it to possess antinociceptive activity similar to that of paracetamol or 4-aminophenol. HMBA (100 mg/kg) inhibited both the first and second phases of the formalin test in wild-type mice (Fig. 6A), but affected none of these phases in FAAH*^−/−^* mice in contrast to their wild-type littermates (Fig. 6B).

It was recently shown that the analgesic dipyrone is also subjected to a FAAH-dependent metabolic conversion to bioactive N-arachidonoylamines that accumulate in the mouse CNS after repeated administration [13]. One of these metabolites behaved as a weak blocker of TRPV1-mediated calcium responses *in vitro* with an IC_50_ of approximately 3 µM [14]. We found that dipyrone (50 mg/kg i.p.) is an effective antinociceptive agent in the mouse formalin test and that this action is independent of FAAH, as the compound produced similar effects in FAAH*^−/−^* mice and their wild-type littermates (Fig. 6C).

#### Involvement of TRPV1

**Figure 7:**
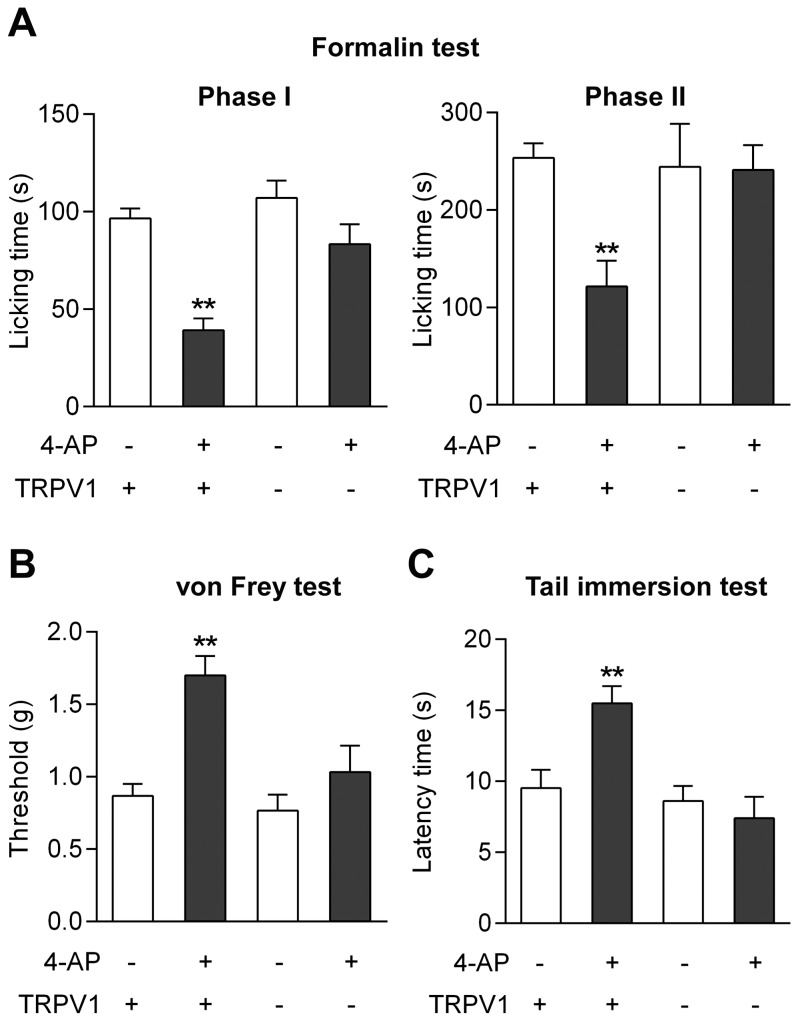
The antinociceptive effect of 4-aminophenol is dependent on TRPV1 in the mouse. The antinociceptive effect of 4-aminophenol (30 mg/kg) in the mouse formalin (**A**; n = 5–6), von Frey (**B**; n = 6) and tail immersion (**C**; n = 6) tests disappeared in TRPV1^−/−^ mice as compared to their wild-type littermates. 4-Aminophenol was injected by the intraperitoneal route 10 min before the tests. **p<0.01 when compared to vehicle injection, using Mann-Whitney *U* test.

**Figure 8:**
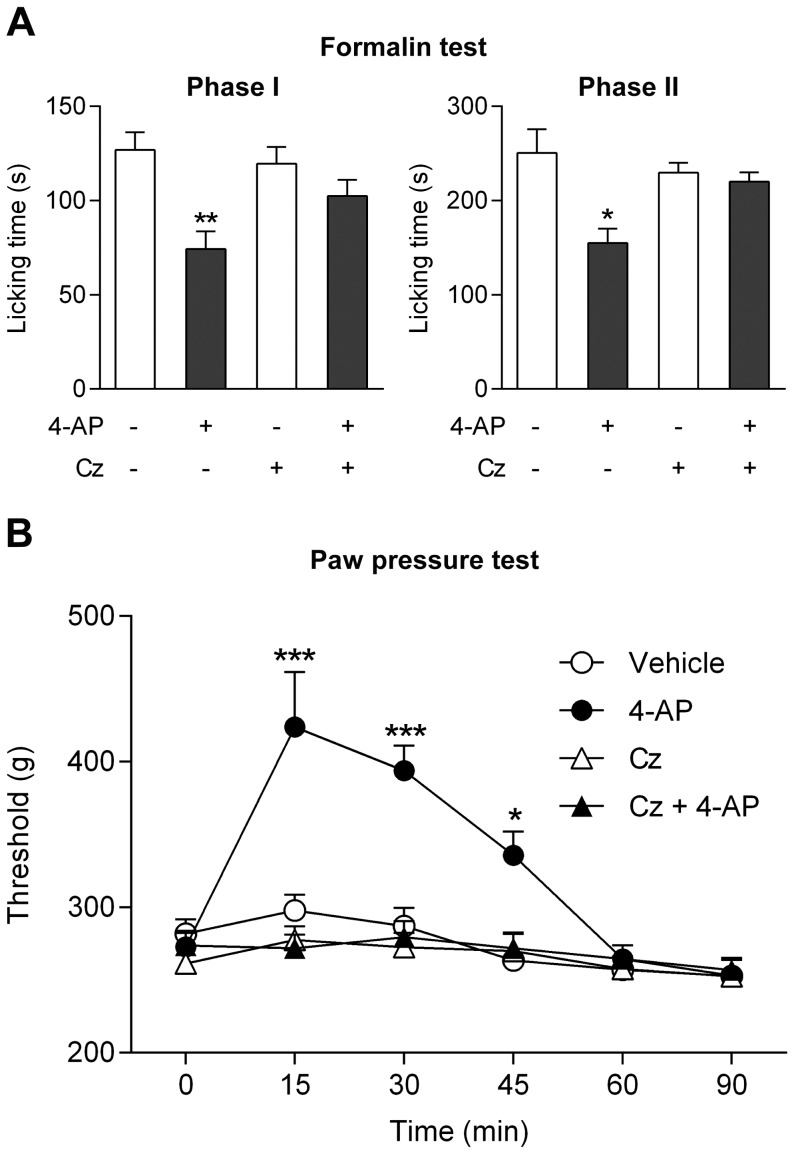
The antinociceptive effect of 4-aminophenol is dependent on TRPV1 in the rat. The TRPV1 blocker capsazepine (10 mg/kg) prevented the antinociceptive effect of 4-aminophenol (100 mg/kg) in the rat formalin (**A**; n = 7–10) and paw pressure (**B**; n = 6–8) tests. Capsazepine (Cz) and 4-aminophenol were injected by the intraperitoneal route 30 min and 10 min before the tests, respectively. *p<0.05, **p<0.01 and ***p<0.001 when compared to vehicle injection, using Mann-Whitney *U* test (**A**) or repeated measures two-way ANOVA followed by Sidak’s multiple comparisons test (**B**).

We have previously reported that genetic inactivation of TRPV1 abolishes the antinociceptive effects of paracetamol in the mouse formalin, von Frey and tail immersion tests [3]. Here we show that these findings extend to 4-aminophenol (30 mg/kg i.p.), the antinociceptive activity of which is also lost in TRPV1^−/−^ mice in these tests (Fig. 7A–C). Furthermore, pretreatment of rats with the TRPV1 blocker capsazepine (10 mg/kg i.p.) prevented the antinociceptive effect of 4-aminophenol in the formalin and paw pressure tests (Fig. 8A and B).

**Figure 9:**
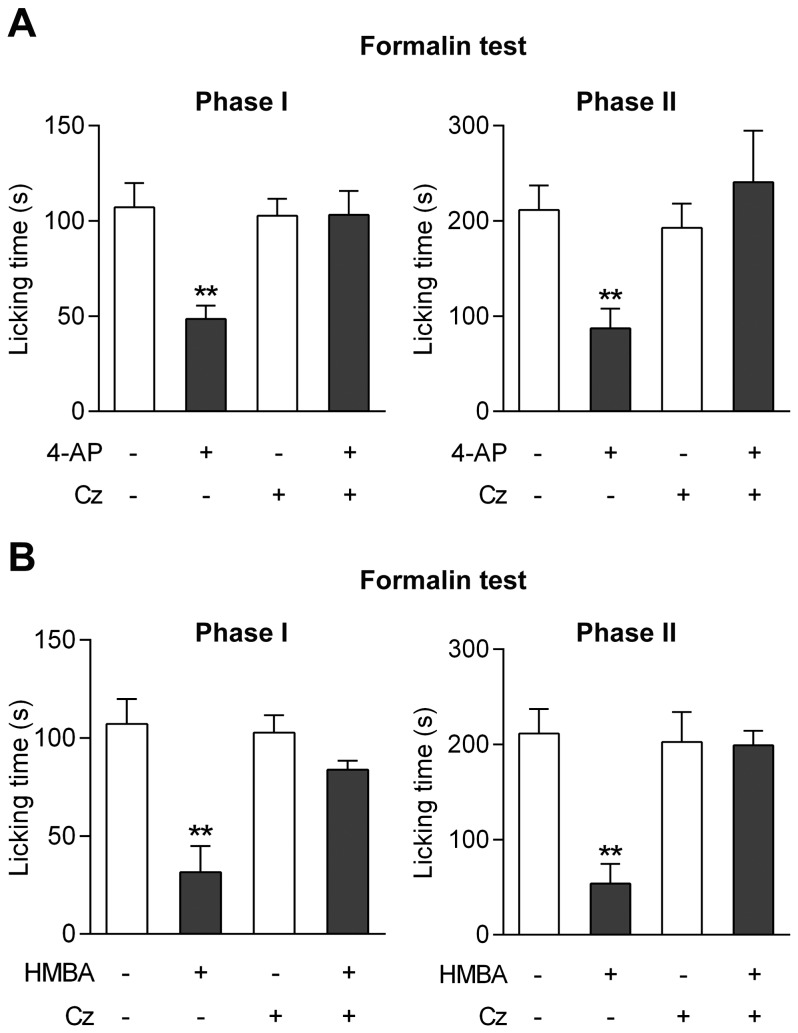
TRPV1 in brain mediates the antinociceptive effects of 4-aminophenol and 4-hydroxy-3-methoxybenzylamine (HMBA). Intracerebroventricular injection of the TRPV1 blocker capsazepine (Cz) prevented the antinociceptive effect of 4-aminophenol (**A**; n = 5–7) and HMBA (**B**; n = 6–8) in the mouse formalin test. 4-aminophenol (30 mg/kg) and HMBA (100 mg/kg) were injected by the intraperitoneal route 10 min before the test. Capsazepine (100 nmol) was injected 5 min before the 4-aminophenol and HMBA administration. **p<0.01 when compared to vehicle injection, using Mann-Whitney *U* test.

These strategies to inactivate TRPV1 do not address the site of action of a drug given systemically. Therefore, to selectively target TRPV1 in the CNS, capsazepine (100 nmol) was injected into the lateral ventricle 5 min prior to 4-aminophenol (30 mg/kg i.p.) or HMBA (100 mg/kg i.p.) administration in mice. Capsazepine eliminated the antinociceptive effects of 4-aminophenol and HMBA in the mouse formalin test, rendering the drugs inactive on both phases of the test (Fig. 9A and B). Inspection of the brain and the thoracic and lumbar spinal cord after methylene blue injection demonstrated that staining was confined to brain tissue surrounding the cerebral ventricles (n = 4).

### Further Evidence for a Similar Pharmacological Profile of 4-aminophenol and Paracetamol

#### Involvement of cannabinoid CB1 receptors

**Figure 10:**
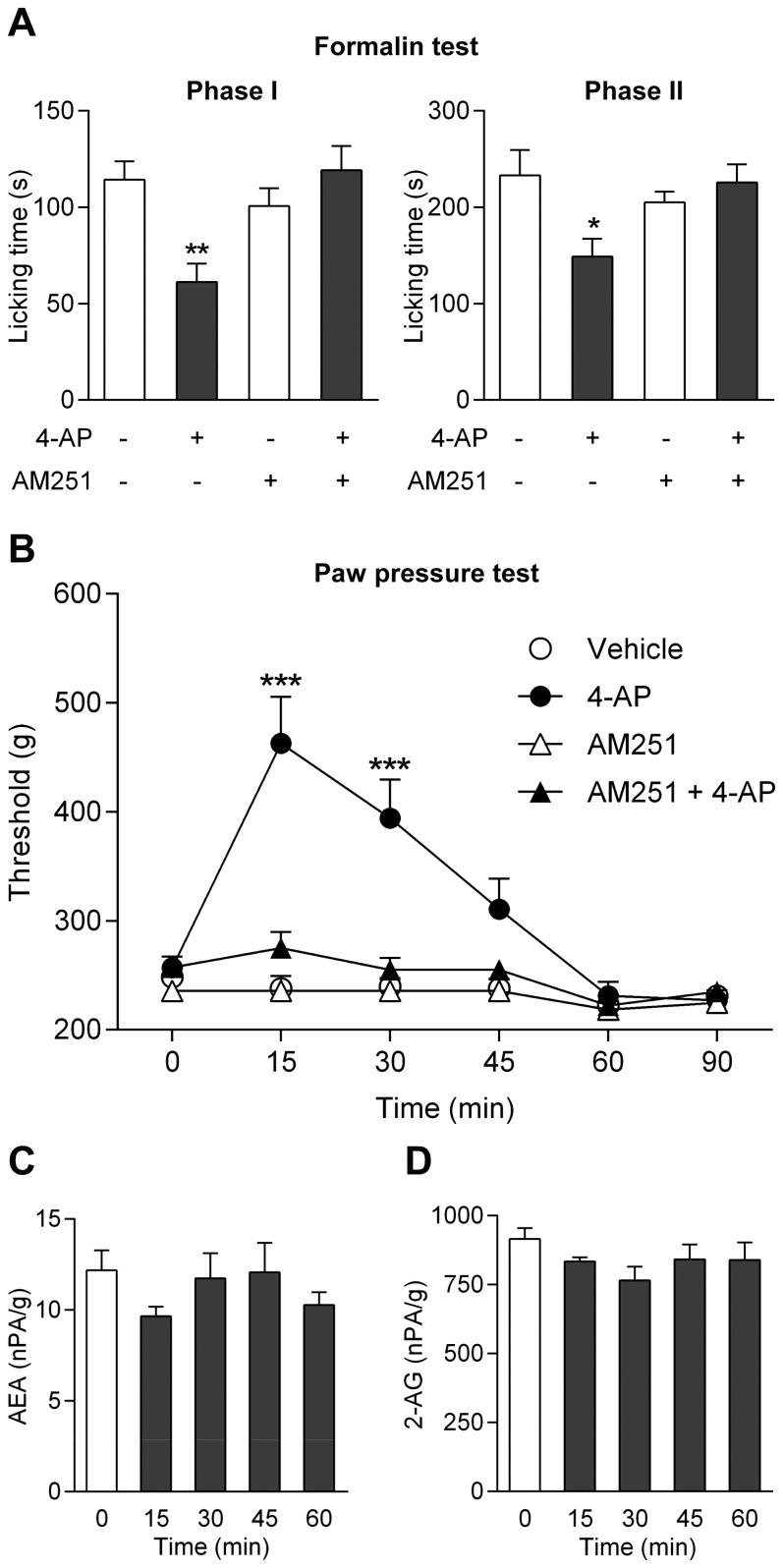
The antinociceptive effect of 4-aminophenol is dependent on cannabinoid CB1 receptors. The cannabinoid CB1 receptor antagonist AM251 (3 mg/kg) prevented the antinociceptive effect of 4-aminophenol (100 mg/kg) in the rat formalin (**A**; n = 8–11) and paw pressure (**B**; n = 6–7) tests. However, 4-aminophenol (100 mg/kg) did not affect the global brain levels of the endocannabinoids anandamide (**C**; n = 6) and 2-arachidonoylglycerol (2-AG; **D**; n = 6). AM251 and 4-aminophenol were injected by the intraperitoneal route 30 min and 10 min before the behavioural assays, respectively. The contents of endocannabinoids are presented as normalized peak area (nPA) per g protein. *p<0.05, **p<0.01 and ***p<0.001 when compared to vehicle injection, using Mann-Whitney *U* test (**A**), repeated measures two-way ANOVA followed by Sidak’s multiple comparisons test (**B**) or Kruskal-Wallis one-way ANOVA followed by Dunn’s multiple comparisons test (**C** and **D**).

It is intriguing that the antinociceptive effect of paracetamol in, e.g., the rat formalin and paw pressure tests is inhibited by the cannabinoid CB1 receptor antagonist AM251 [12]. Again, we find that observations made on paracetamol can be extended to 4-aminophenol, as pretreatment with the cannabinoid CB1 receptor antagonist AM251 (3 mg/kg i.p.) prevented the antinociceptive effect of 4-aminophenol (100 mg i.p.) in the rat formalin and paw pressure tests (Fig. 10A and B). Notably, this dose of 4-aminophenol did not affect the global levels of the endocannabinoids anandamide and 2-arachidonoylglycerol in the rat brain 15, 30, 45 and 60 min after 4-aminophenol administration (Fig. 10C and D), as previously shown for paracetamol [2].

#### Involvement of spinal serotonergic mechanisms

**Figure 11:**
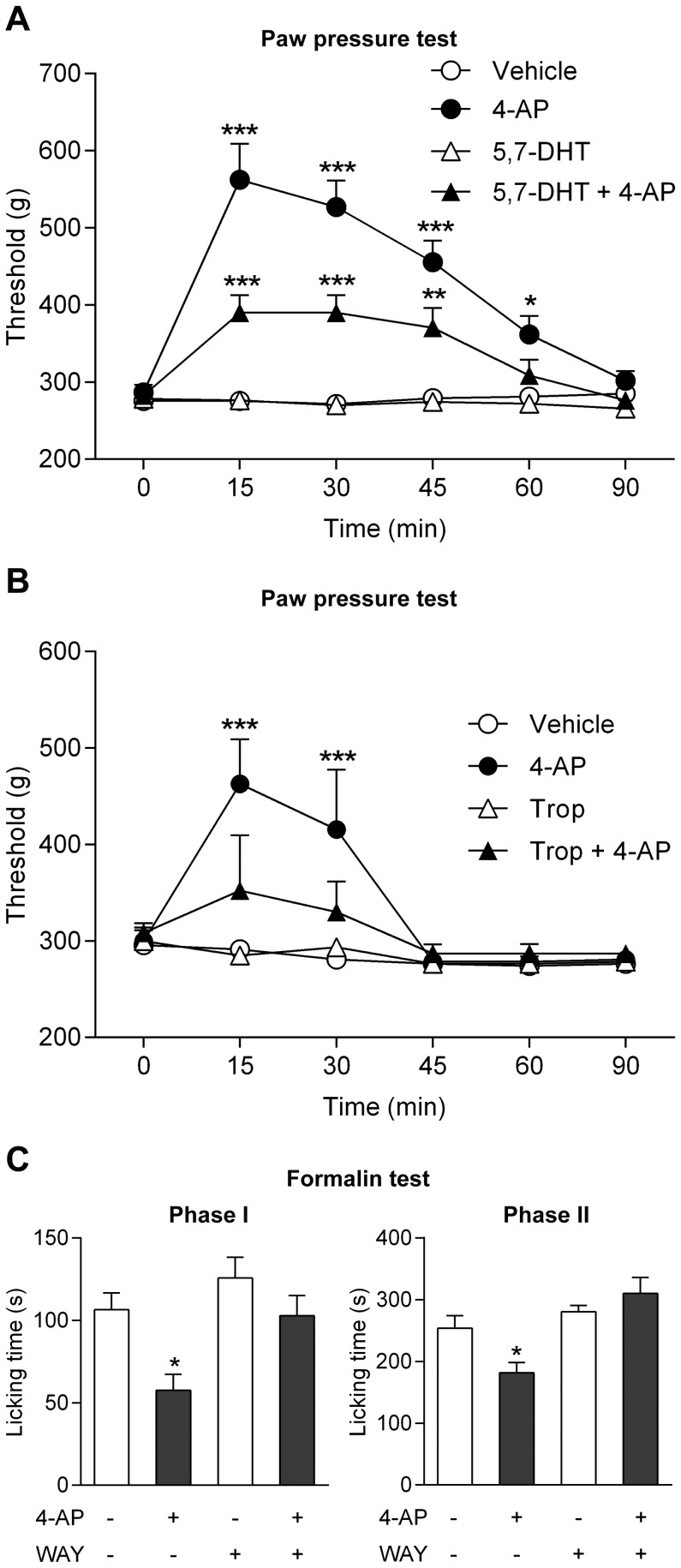
The antinociceptive effect of 4-aminophenol is dependent on spinal serotonergic mechanisms. Pre-treatment of rats with the neurotoxin 5,7-dihydroxytryptamine (5,7-DHT) reduced the 4-aminophenol-induced increase of the withdrawal threshold in the paw pressure test (**A**; n = 7–8). The serotonin receptor antagonists tropisetron (**B**; n = 7–8) and WAY-100635 (**C**; n = 6–9) prevented the antinociceptive effect of 4-aminophenol in the rat paw pressure and formalin tests, respectively. 5,7-DHT (100 µg) was administered intrathecally 7 days before the paw pressure test. 4-Aminophenol (100 mg/kg) was injected by the intraperitoneal route 10 min before the behavioural tests. Tropisetron (Trop; 500 ng) and WAY-100635 (WAY; 40 µg) were injected intrathecally 5 min before administration of 4-aminophenol. *p<0.05, **p<0.01 and ***p<0.001 when compared to vehicle injection, using repeated measures two-way ANOVA followed by Sidak’s multiple comparisons test (**A** and **B**) or Mann-Whitney *U* test (**C**).

Studies in both animals and man have implicated an important role of bulbospinal pathways and serotonergic mechanisms in the analgesic effect of paracetamol [12], [15]–[17]. We therefore explored whether spinal serotonergic mechanisms are also involved in the antinociceptive effect of 4-aminophenol. The neurotoxin 5,7-dihydroxytryptamine (5,7-DHT) was injected intrathecally (100 µg/rat) to produce lesion of spinal serotonergic pathways in the rat. This treatment reduced the spinal serotonin content from 529±34 to 359±38 ng/g tissue wet weight (p<0.05) and inhibited the antinociceptive effect of 4-aminophenol, as measured 7 days after 5,7-DHT treatment (Fig. 11A). Furthermore, intrathecal injection of the 5-HT_3_ receptor antagonist tropisetron (500 ng/rat) and the selective 5-HT_1A_ receptor antagonist WAY-100635 (40 µg/rat) 5 min before administration of 4-aminophenol (100 mg/kg i.p.) prevented, as for paracetamol [12], the antinociceptive effect in the rat paw pressure and formalin tests, respectively (Fig. 11B and C).

## Discussion

**Figure 12:**
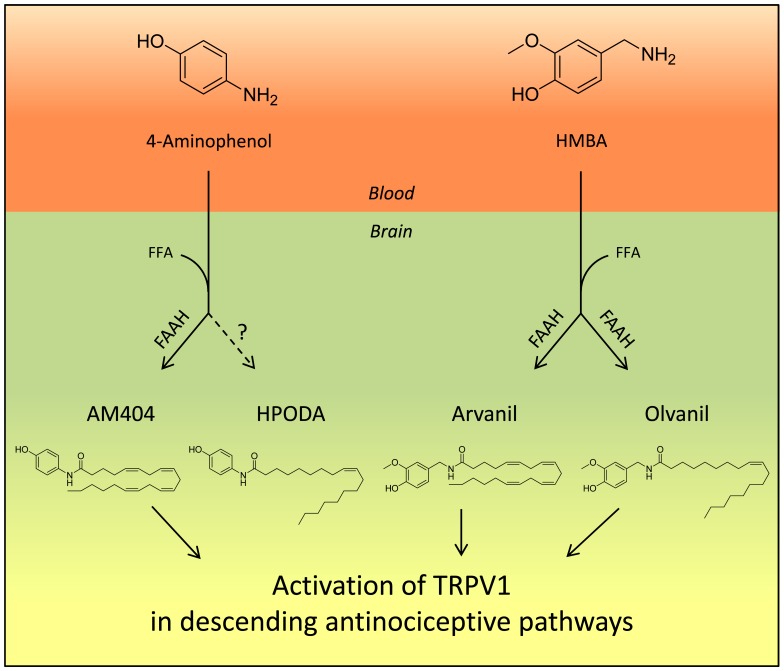
Proposed mechanism by which 4-aminophenol and 4-hydroxy-3-methoxybenzylamine (HMBA) produce antinociception. Following passage through the blood-brain barrier, 4-aminophenol and HMBA are conjugated with free fatty acids (FFA), preferentially arachidonic acid, to yield the potent TRPV1 activators AM404 and arvanil plus olvanil, respectively, a reaction catalyzed by the enzyme fatty acid amide hydrolase (FAAH). AM404, arvanil and olvanil then activate TRPV1 in brain nuclei, regulating descending antinociceptive pathways, possibly of serotoninergic origin, thereby reducing nociceptive neurotransmission in the dorsal horn of the spinal cord. In contrast to the other lipid metabolites, HPODA is produced by a FAAH-independent pathway and it does not seem to contribute to the antinociceptive action of 4-aminophenol, consistent with HPODA being produced in smaller quantities and being a less potent TRPV1 activator than AM404.

The staggering literature on paracetamol provides a complex picture with several metabolites and mechanisms potentially contributing to paracetamoĺs analgesic activity, including TRPV1 activation in the brain by AM404 and possibly other N-4-(hydroxyphenyl)acylamide metabolites of paracetamol [2], [3], recruitment of bulbospinal serotonergic pathways [12], [15]–[17], activation of spinal TRPA1 by electrophilic paracetamol metabolites [18] and inhibition of prostaglandin production [19]. In the present study, we show that systemic administration of 4-aminophenol and HMBA generates potent TRPV1 active drug metabolites in the mouse brain and produces antinociception in various pain tests, including the mouse formalin (4-aminophenol, HMBA), von Frey (4-aminophenol) and tail immersion (4-aminophenol) tests, and the rat formalin (4-aminophenol) and paw pressure (4-aminophenol) tests. The brain levels of the TRPV1 active drug metabolites, except HPODA, were substantially reduced in FAAH^−/−^ mice and the antinociceptive activities of 4-aminophenol and HMBA disappeared in FAAH^−/−^ and TRPV1^−/−^ mice. Similar results were obtained in rats subjected to pharmacological inhibition of FAAH and TRPV1. It is unlikely that inhibition of prostaglandin synthesis contributed to the antinociceptive effects of 4-aminophenol and HMBA, because the mouse tests used, except for the second phase of the formalin test, reflect non-inflammatory acute pain that is independent of COX [3]. These findings support our proposal that activation of TRPV1 by FAAH-dependent drug metabolites underlies the antinociceptive activity of 4-aminophenol and HMBA in these tests, as has been shown for paracetamol (Fig. 12) [3]. The similarities between the antinociceptive activities of 4-aminophenol and paracetamol in terms of their dependence on FAAH, TRPV1, cannabinoid CB1 receptors and spinal serotonergic mechanisms provide additional evidence that 4-aminophenol is the key intermediate metabolite in the bioactivation of paracetamol.

We show that 4-aminophenol and HMBA given systemically produced a dose-dependent formation of AM404 plus HPODA and arvanil plus olvanil in the mouse brain, respectively, and that intracerebroventricular injection of the TRPV1 blocker capsazepine prevented the antinociceptive effects of 4-aminophenol and HMBA in the mouse formalin test. We could not detect any staining of the thoracic and lumbar spinal cord after intracerebroventricular injection of the water soluble dye methylene blue. Thus, it is unlikely that capsazepine directly interfered with the primary afferent input to the dorsal horn of the spinal cord following formalin injection. However, we cannot exclude an interaction with TRPV1 on central projections of primary afferents terminating in the trigeminal or solitary tract nuclei. Taken together, our findings indicate that activation of TRPV1 in brain plays a key role in the antinociceptive effects of 4-aminophenol and HMBA and suggest that lipid metabolites, such as AM404 and arvanil, mediate these actions.

As shown previously, intracerebroventricular injection of AM404 can reproduce the TRPV1-dependent antinociceptive effect of oral paracetamol in the mouse formalin test [3], although the duration of action of AM404 was relatively short, probably reflecting rapid elimination of AM404 in the absence of its precursor 4-aminophenol [20]. Arvanil given by the same route also possesses antinociceptive activity, as shown in the mouse tail-flick test [21]. However, the brain contents of arvanil (3.33 pmol/g) and olvanil (below the level of quantification) were much lower than the content of AM404 (411 pmol/g) and HPODA (96 pmol/g) after systemic administration of HMBA (100 mg/kg) and 4-aminophenol (30 mg/kg), respectively. This does not necessarily mean that arvanil and olvanil produced less TRPV1 activation than AM404 and HPODA, because the final responses also depend on the potencies of the compounds. Interestingly, arvanil was shown to be equipotent with the ultrapotent TRPV1 activator resiniferatoxin in functional assays and radioligand binding studies of rodent TRPV1 [9], [11]. We have identified two previous studies that directly compare the TRPV1 activity of AM404 with that of arvanil or olvanil. One study reported that arvanil was 933 times more potent than AM404 in a ^45^Ca^2+^ uptake assay, using CHO cells expressing the rat orthologue of TRPV1 [11]. In another study, arvanil and olvanil were approximately 50 times more potent than AM404 in HEK293 cells expressing the human TRPV1, using fluorometric calcium imaging [10]. In the present study, we found that arvanil and olvanil were >100 times more potent than AM404 and >300 times more potent than HPODA as TRPV1 activators. Taking these potency differences into account, it is likely that the amounts of AM404 plus HPODA and arvanil plus olvanil formed after 4-aminophenol (30 mg/kg) and HMBA (100 mg/kg) administration, respectively, produced comparable TRPV1 activation in the mouse brain. Indeed, these doses of HMBA and 4-aminophenol produced equivalent antinociception in the mouse formalin test.

The contribution of HPODA to the antinociceptive effect of 4-AP is, however, less clear. This N-acylamine was not only less potent than AM404 as a TRPV1 activator, but its level in the mouse brain after 4-aminophenol administration was approximately one fourth of that of AM404. More importantly, while the antinociceptive activity of 4-aminophenol (30 mg/kg) disappeared in FAAH^−/−^ mice, the brain content of HPODA after this dose of 4-aminophenol was 3 times larger in these mice than in their wild-type littermates. Thus, in contrast to the oleic acid derivative of HMBA (olvanil), the corresponding derivative of 4-aminophenol (HPODA) seems to be generated mainly by a FAAH-independent biosynthetic pathway, although FAAH may mediate the degradation of both these N-acylamines. These findings indicate not only quantitative differences, but also qualitative differences in the fatty acid conjugation of 4-aminophenol and HMBA.

AM404 was originally introduced as a selective inhibitor of cellular anandamide uptake and degradation [22]–[24]. However, the potency of AM404 as an inhibitor of anandamide degradation in rat brain homogenates is between two and three orders of magnitude lower than the potency of this compound in assays of TRPV1 activity [2], [10], [11]. The selectivity towards TRPV1 is even larger for arvanil and olvanil, which both are weak inhibitors of anandamide uptake and degradation [10], [23], [25]–[27]. This is in line with the global brain levels of endocannabinoids being unaffected by systemic administration of 4-aminophenol (present study) or paracetamol [3]. It is therefore intriguing that the cannabinoid CB1 receptor antagonist AM251 could prevent the antinociceptive effect of 4-aminophenol in the rat formalin and paw pressure tests. We have previously reported similar findings with respect to paracetamol [12]. Unsaturated long chain N-acyl-hydroxyphenylamides and -vanillylamides, including AM404, arvanil and olvanil, are poor cannabinoid receptor agonists [21], [24]–[27]. It is, thus, unlikely that sufficient amounts of AM404 and arvanil plus olvanil are generated from 4-aminophenol and HMBA in the brain, respectively, to sustain a direct activation of the cannabinoid CB1 receptor. A recent study, addressing the intriguing antinociceptive effect of the TRPV1 activator capsaicin in the periaqueductal gray (PAG), an important midbrain region for regulation of descending pain inhibitory pathways projecting to the dorsal horn [28], [29], has provided evidence of the recruitment of cannabinoid CB1 receptors downstream of TRPV1 present on excitatory glutamatergic neurons in the ventrolateral PAG [30]. Microinjection of capsaicin into the rat ventrolateral PAG reduced the nocifensive behaviour in the hot plate test, an effect that was inhibited by co-injection of capsaicin with the cannabinoid CB1 receptor antagonist AM251 as well as with the selective TRPV1 blocker SB 366791 [30]. Patch-clamp recordings in brain slices of the ventrolateral PAG demonstrated that capsaicin produced not only a glutamate-dependent neuronal excitation, but also an endocannabinoid-mediated retrograde inhibition of GABAergic neurons, an effect reversed by AM251 [30]. Such a dual effect of TRPV1 activators in the ventrolateral PAG may explain the seemingly contradictory finding that pharmacological or genetic inactivation of the cannabinoid CB1 receptor is able to inhibit the antinociceptive effects 4-aminophenol and paracetamol.

Bulbospinal serotonergic pathways originate in several brainstem nuclei, including the nucleus raphe magnus. These pathways receive input from PAG and contribute to the descending regulation of nociceptive signalling via serotonin release and activation of its cognate receptors on dorsal horn neurons [31]. Our findings that intervention with spinal serotoninergic mechanisms prevents the antinociceptive effects of 4-aminophenol (present study) and paracetamol [12] provide additional evidence that supraspinal TRPV1 may regulate nociceptive neurotransmission via activation of descending bulbospinal pathways [32]. TRPV1 activation also facilitates glutamate neurotransmission in the dorsolateral PAG and in other brain areas of potential importance for the regulation of descending pain inhibition, including the locus coeruleus and paraventricular nucleus [33]–[36]. Thus, the identity and localization of the TRPV1-expressing neurons activated by the lipids metabolites of paracetamol, 4-aminophenol and HMBA, and the relationship between these neurons and the descending bulbospinal serotonergic pathways remain to be elucidated.

Several factors may influence the biotransformation of 4-aminophenol and HMBA to TRPV1 active lipid metabolites in the brain, including drug absorption from the site of injection, distribution of the drug to the brain and FAAH-dependent fatty acid conjugation to yield the various bioactive metabolites. Our pharmacokinetic findings show that relatively small changes in the chemical structure may have profound effects on the propensity of the molecule to undergo fatty acid conjugation. Indeed, mice receiving equieffective doses of 4-aminophenol (30 mg/kg) and HMBA (100 mg/kg) in the mouse formalin test produced similar brain contents of 4-aminophenol (91 nmol/g) and HMBA (132 nmol/g). However, the corresponding brain contents of AM404 and arvanil were 411 pmol/l and 3.33 pmol/l, respectively, indicating that the FAAH-mediated conjugation with arachidonic acid was approximately 100 times more efficient for 4-aminophenol than HMBA, two structurally similar primary amines (Fig. 12).

The hepatotoxicity of 4-aminophenol is very low in rodents and may involve its N-acetylation to paracetamol [37], [38]. However, 4-aminophenol has been shown to produce renal tubular necrosis after a single exposure at doses not far from those eliciting antinociception in rodents [39]–[41]. This selective nephrotoxic effect seems to depend on the extrarenal formation of toxic glutathione conjugates of 4-aminophenol, including 4-amino-3-cysteinylphenol, which then accumulates in proximal tubular epithelial cells, although a direct cytotoxic effect of 4-aminophenol on tubular epithelial cells cannot be excluded [42], [43]. The glutathione transferase capacity differs greatly between species and it is particularly high in rats and mice, making predictions of tolerable doses in man very difficult [44]. In contrast to 4-aminophenol and paracetamol, HMBA should not generate reactive benzoquinone imines and therefore may display low nephro- and hepatotoxicity. Further animal studies addressing the toxicology and the antinociceptive activity in models of inflammatory pain are warranted before considering 4-aminophenol and HMBA as potential analgesics for parenteral use in man.

This study suggests that FAAH-dependent generation of antinociceptive TRPV1 active drug metabolites in the brain represent a novel pharmacological principle for treatment of pain and strengthens that 4-aminophenol is the key FAAH substrate in the bioactivation of paracetamol.

## Materials and Methods

### Ethics Statement

The animal procedures were approved by the animal ethics committees in Auvergne, France (CEMEA Auvergne, No. CE3–06) and Lund, Sweden (Malmö/Lunds djurförsöksetiska nämnd, No. M 111-10). The behavioural tests were conducted in accordance with the official edict presented by the French Ministry of Agriculture (Paris), the European Community Council Directive (86/609/EEC) and the International Association for the Study of Pain (IASP) guidelines for animal experiments [45].

### Animals

Experiments were performed on adult C57BL/6 mice (20–30 g) and Sprague-Dawley rats (175–225 g) from Charles River Laboratories (Lyon, France) or Taconic (Denmark). Only male animals were used in the behavioral tests. TRPV1^−/−^ and FAAH^−/−^ mice were originally generated by David Julius [46] and Benjamin Cravatt [47], respectively. These mice were back-crossed with C57BL/6 mice for at least ten generations and their genotype confirmed by polymerase chain reaction. Animals were housed under controlled environmental conditions (21–22°C; 55% humidity) and kept under a 12/12 h light/dark cycle with food and water *ad libitum* for at least one week prior to the start the experiments.

### Assessment of TRPV1 Activity

TRPV1 activation was assessed indirectly on rat isolated mesenteric arteries, using nerve-mediated vasorelaxation as readout [2]. Briefly, arterial segments (2 mm long) were suspended between two stainless steel wires in temperature-controlled tissue baths (37°C), containing aerated (95% O_2_ and 5% CO_2_) physiological salt solution (composition in mM: NaCl 119, KCl 4.6, CaCl_2_ 1.5, MgCl_2_ 1.2, NaHCO_3_ 15, NaH_2_PO_4_ 1.2 and D-glucose 6; pH 7.4), under a passive load of 2 mN. One of the wires was connected to a force-displacement transducer model FT03 C (Grass Instruments; Rhode Island, USA) for isometric tension recording. Vasorelaxation was studied in arterial segments submaximally contracted with phenylephrine. Increasing concentrations of AM404, arvanil, olvanil and HPODA in the absence and presence of capsazepine (3 µM) were then added cumulatively to determine concentration-response relationships. All experiments were performed in the presence of indomethacin (10 µM) and N^ω^-nitro-L-arginine (300 µM) to reduce cyclooxygenase and nitric oxide synthase activity, respectively. Under these conditions, phenylephrine induces stable and long-lasting contractions, which were minimally affected by the vehicle used.

### Biochemical Studies

#### Experiments on brain homogenates *in vitro*

Rat brain homogenate were prepared, as previously described [2]. Brains were homogenized in Tris buffer (10 mM, pH 7.6), containing ethylenediaminetetraacetic acid (EDTA; 1 mM), at volumes of 5–10 ml/g tissue. Experiments were carried out on aliquots of 200 µl homogenate at 37°C as further explained in the text. The reactions were stopped by adding 1 ml ice-cold acetone, containing 1 µM [^2^H_8_]-anandamide. The samples were kept on ice until the acetone phase was vacuum evaporated.

#### *In vivo* experiments

4-Aminophenol (30 and 100 mg/kg) and HMBA (100 and 300 mg/kg) were administered to mice and rats by intraperitoneal injections in volumes of 10 ml/kg. Twenty min after drug administration, the animals were anesthetized by CO_2_ (rats) or isoflurane (mice) inhalation and decapitated. The brain was removed from the skull and blood was collected in test tubes, containing 60 µl buffered citrate, and the tissues snap frozen in liquid nitrogen before storage at −70°C.

The whole brain was homogenized in ice-cold 10 mM Tris buffer (ml/g tissue) at pH 7.6. The buffer solution contained 1 mM EDTA, 0.3 mM ascorbic acid and 10 µM methylarachidonylfluorophosphonate (MAFP) to prevent degradation of AM404, arvanil, olvanil, anandamide and 2-arachidonoylglycerol. Aliquots (200 µl) of blood and homogenates were precipitated with 1 ml ice-cold acetone, containing AM404-D_4_ (5 nM) or anandamide-D_8_ (100 nM) as internal standard. After centrifugation at 25,200 g for 30 min at 4°C, the supernatants were collected in polypropylene tubes and vacuum evaporated. The protein content of the pellet was determined with Coomassie protein assay (Pierce; Illinois, USA), using bovine serum albumin as standard. The extraction residues were reconstituted in 100 µl methanol (AM404, arvanil, olvanil anandamide and 2-arachidonoylglycerol) or 0.5% acetic acid (4-aminophenol and HMBA). Samples were analyzed on a Waters Aquity UPLC system coupled to a Sciex 5500 tandem mass spectrometer (LC-MS/MS; AB Sciex; Massachusetts, USA) or a Perkin Elmer 200 HPLC system coupled to an API 3000 tandem mass spectrometer (LC-MS-MS; Applied Biosystems/MDS-SCIEX).

#### Mass spectrometry

*Quantification of AM404, HPODA, arvanil and olvanil.* Samples were analyzed on a Waters Aquity UPLC system coupled to a Sciex 5500 tandem mass spectrometer. Sample aliquots of 5 µl were injected into an Acquity UPLC BEH C_18_ column (100 × 2 mm, 1.7 µm; Waters) held at 50°C. All mobile phases were water-acetonitrile gradients, containing 0.1% formic acid and using a flow rate of 400 µl/min. The acetonitrile content was initially 30%, then increased linearly to 100% over 7 min and kept at this level for 4 min, followed by an equilibration period at 30% for 2 min. The electrospray interface was operating in the positive ion mode at 550°C and the ion spray voltage was set to 5000 volts. The mass transitions (m/z) used were the following: 396.2/110.1 for AM404, 374.2/110.0 for HPODA, 400.2/114.0 for AM404-D_4_, 440.3/137.1 for arvanil and 418.3/137.1 for olvanil. The declustering potentials were between 96 and 130 volts and the collision energies between 25 and 41 volts.

*Quantification of 4-aminophenol and HMBA.* Sample aliquots of 5 µl were injected into a Hypercarb column (50 × 2.1 mm; Thermo). All mobile phases were water-acetonitrile gradients, containing 0.1% formic acid. The acetonitrile content was initially 2%, then increased linearly to 75% over 5 min and then kept at 100% for 1 min, followed by an equilibration period at 2% for 1.5 min. The electrospray ion source was set at 550°C and used in the positive ion mode with an ion spray voltage of 5000. The mass transitions (m/z) was 110.1/93.0 for 4-aminophenol. HMBA loses the amino group already in the source of the MS and therefore the mass transition used was 137.0/94.0. The declustering potentials were 100 and 90 volts and the collision energies 21 and 29 volts. No internal standard was used for these analyses.

*Quantification of anandamide and 2-arachidonoylglycerol*. Anandamide (m/z 348.2/62.0), anandamide-D_8_ (m/z 356.4/63.0) and 2-arachidonoylglycerol (m/z 379.2/287.0) were determined as previously described [3]. Since 2-arachidonoylglycerol is non-enzymatically converted to 1-arachidonoylglycerol, the content of 2-arachidonoylglycerol was estimated as the sum of these lipids.

The contents of the compounds were expressed either in moles or as normalized peak areas (nPA) and related to the protein content in the samples. The nPA was obtained by dividing the peak area of the analyte with the peak area for the internal standard in the same sample. The detection limits were calculated as the concentrations corresponding to three times the standard deviation of the blanks. When levels were below detection limit, numerical values of half the detection limit were used in the calculations.

### Behavioural Tests

The behavioural tests were performed in a quiet room and evaluated by a single investigator in a blinded manner. Treatments and/or genotypes were randomized in blocks and the number of animals in each block corresponded to the number of treatments and/or genotypes compared. For example, when the effect of capsazepine on the antinociceptive responses to 4-aminophenol was tested, four animals were included in each block, corresponding to the following treatments: Vehicle+vehicle, vehicle +4-aminophenol, capsazepine+vehicle and capsazepine +4-aminophenol. Each animal was exposed to only one treatment. All behavioral tests except the rat paw pressure test were performed 10 min after the intraperitoneal injection of 4-aminophenol, HMBA, dipyrone or vehicle. PMSF (s.c.) and capsazepine (i.p.) were administrated 20 min and 10 min before 4-aminophenol (i.p.), respectively. Capsazepine (i.c.v.), tropisetron (i.t.) and WAY-100635 (i.t.) were injected 5 min before 4-aminophenol (i.p.) or HMBA (i.p.).

#### Assessment of locomotor activity – mouse

Mice were placed in actimetry boxes (Actisystem; Apelex, France) and spontaneous motor activity was assessed by determining the number of crossings of light beams during 15 min.

#### Formalin test – mouse and rat

The animals received an intraplantar injection of a 2.5% formalin solution (50 µl rat, 25 µl mouse) into a hind paw. The time spent biting and licking of the injected paw was monitored during the two typical phases of the nociceptive response (phase I: 0–5 min; phase II: 20–40 min in rats or 15–40 min in mice).

#### Paw pressure test – rat

The rats were submitted to the paw pressure test, using an Ugo Basile analgesy meter with a probe tip diameter of 1 mm (Bioseb, France). Nociceptive thresholds, expressed in grams (g), were measured by applying an increasing pressure to the right hind paw until a squeak (vocalization thresholds) was obtained (cut-off 750 g). Two consecutive stable vocalisation threshold values were first obtained (baseline). The various treatments were then given and the measurements repeated 15, 30, 45, 60 and 90 after 4-aminophenol or vehicle administration.

#### von Frey filament test – mouse

Calibrated von Frey filaments (0.0045–5.4950 g) were used to induce light noxious mechanical stimulation in mice [48]. Tests were commenced after one hour of habituation. Filaments of increasing stiffness were applied five times perpendicular to the plantar surface of the hindpaw and pressed until bending. The first filament that evoked at least three consecutive responses was assigned as the threshold (cut-off 2 g).

#### Tail immersion test – mouse

Tails of mice were submerged in a hot water bath (46°C) and the withdrawal latency recorded (cut-off 15 s). The mean of four baseline values were determined before drug administration.

#### Intracerebroventricular (mouse) and intrathecal (rat) injections

Capsazepine (100 nmol) and methylene blue (20 µg) were injected into the lateral ventricle of mice, using a 25 µl Hamilton syringe. The injection site was 1 mm lateral to the midline drawn through the anterior base of the ears. The syringe was inserted perpendicularly through the skull into the brain to a depth of 4 mm, where 2 µl of the solution was injected, as previously described [3]. Mice were sacrificed 45 min after methylene blue injection and the brain and the thoracic and lumbar spinal cord were removed for ocular inspection. Intrathecal injections were performed on anaesthetised rats held in one hand by the pelvic girdle. A 25 gauge 1 inch needle connected to a 25 µl Hamilton syringe was then inserted into the subarachnoidal space between lumbar vertebrae L5 and L6 until a tail flick was elicited. The syringe was held in position for few seconds after the injection of a volume of 10 µl [49]. All injections were performed under isoflurane anaesthesia (4% induction, 2% maintenance).

#### Lesion of the descending serotonergic pathways – rat

5,7-Dihydroxytryptamine (5,7-DHT; 100 µg/rat) was administered intrathecally 7 days before the experiment. Desipramine (10 mg/kg) was administered (i.p.) 30 min before 5,7-DHT to prevent the uptake of 5,7-DHT by monoaminergic neurons. After the experiment, the animal was sacrificed and the lumbar spinal cords collected for quantification of serotonin by HPLC [12]. Briefly, the dorsal horn of the lumbar spinal cord was homogenized in a saturated KCl solution and centrifuged at 15,000 g for 15 min. The resulting supernatants were mixed with 400 µl of an internal standard (*n*-methyl-serotonin 0.6 mg/l, diluted in glycine buffer at pH 11) and extracted with 2.5 ml of dichloromethane/butanol (75/25). The organic layer was back-extracted with 300 µl of 0.1 M phosphate buffer (pH 4.4). One-hundred µl of this solution was injected in the HPLC system with an electrochemical detector.

### Drugs

4-Aminophenol, 4-hydroxy-3-methoxybenzylamine (HMBA), phenylmethylsulfonyl fluoride (PMSF), *N*-[2-(4-Chlorophenyl)ethyl]-1,3,4,5-tetrahydro-7,8-dihydroxy-2*H*-2-benzazepine-2-carbothioamide (capsazepine), *N*-[2-[4-(2-methoxyphenyl)-1-piperazinyl]ethyl]-*N*-2-pyridinylcyclohexane-carboxamide maleate (WAY-100635), 5,7-dihydroxytryptamine (5,7-DHT) and desipramine were purchased from Sigma-Aldrich (France). N-(4-Hydroxyphenyl)-5Z,8Z,11Z,14Z-eicosatetraenamide (AM404), N-(4-hydroxy-3-methoxybenyl)-9Z-octadecenamide (olvanil), anandamide, 2-arachidonoylglycerol and *N*-(piperidin-1-yl)-5-(4-iodophenyl)-1-(2,4-dichlorophenyl)-4-methyl-1*H*-pyrazole-3-carboxamide (AM251) were obtained from Tocris Cookson (UK). N-(4-hydroxy-3-methoxybenzyl)-5Z,8Z,11Z,14Z-eicosatetraenamide (arvanil) was purchased from Cayman Chemicals (USA). Tropisetron was a gift from Novartis (France) and N-(4-hydroxyphenyl)-9Z-octadecenamide (HPODA) was provided by Prof. Olov Sterner (Lund University, Lund, Sweden). AM404, arvanil, olvanil, and HPODA were dissolved in ethanol. PMSF and AM251 were dissolved in DMSO. Capsazepine was dissolved in ethanol (*in vitro* tests), DMSO (intraperitoneal injection) or physiological serum/DMSO/Tween 80 (85/10/5; intracerebroventricular injection). 4-Aminophenol was dissolved in physiological saline (mouse) or DMSO (rat). HMBA, tropisetron, 5,7-DHT and desipramine were dissolved in physiological saline (0.9% NaCl). The 5,7-DHT solution contained 0.2 mg/ml ascorbic acid. Intraperitoneal and subcutaneous injections were given in volumes of 5–10 ml/kg.

### Calculations and Statistical Analysis

Data are presented as the mean ± SEM, and *n* indicates the number of animal tested in each group. pEC_50_ of the lipid metabolites indicates the negative log concentration that elicited half-maximal vasorelaxation. Mann-Whitney *U* test or Student’s *t*-test was used when two groups were compared. When multiple groups were compared, Kruskal-Wallis one-way ANOVA followed by Dunn’s multiple comparisons test was used. Data from the paw pressure test were analyzed by repeated measures two-way ANOVA followed by Sidak’s or Dunnett’s multiple comparisons test. The level of statistical significance was set at p<0.05. GraphPad Prism 6.0 software (California, USA) was used in all calculations and statistical analyses.

## Supporting Information

Figure S1 **Arvanil, olvanil and N-(4-hydroxyphenyl)-9Z-octadecenamide (HPODA) produce TRPV1-dependent vasorelaxation.** The TRPV1 blocker capsazepine significantly suppressed the vasorelaxation evoked by arvanil (p<0.01), olvanil (p<0.01) and HPODA (p<0.001) in rat mesenteric arterial segments precontracted with phenylephrine. Unfilled and filled circles indicate vasodilator responses in the absence (n = 7–12) and presence (n = 6–7) of capsazepine (3 µM), respectively (the former values are the same as in Fig. 1B). Filled triangles indicate responses after pretreatment (60 min) of the vascular segment with capsaicin (1 µM) to inactivate capsaicin-sensitive nerve fibres (n = 6–8). Values are expressed as mean ± SE. Mann-Whitney *U* test was used to compare the areas under the concentration-response curve in the absence and presence of capsazepine. Sigmoidal concentration-response curves (variable slope) were constructed, using GraphPad Prism 6.0 software (California, USA).
(TIF)
