# Supplementary figures and images for: Fatty Acid Amide Hydrolase-Dependent Generation of Antinociceptive Drug Metabolites Acting on TRPV1 in the Brain
Source: PLoS One. 2013 Aug 5;8(8):e70690. doi: 10.1371/journal.pone.0070690 (PMC3734263; doi:10.1371/journal.pone.0070690)

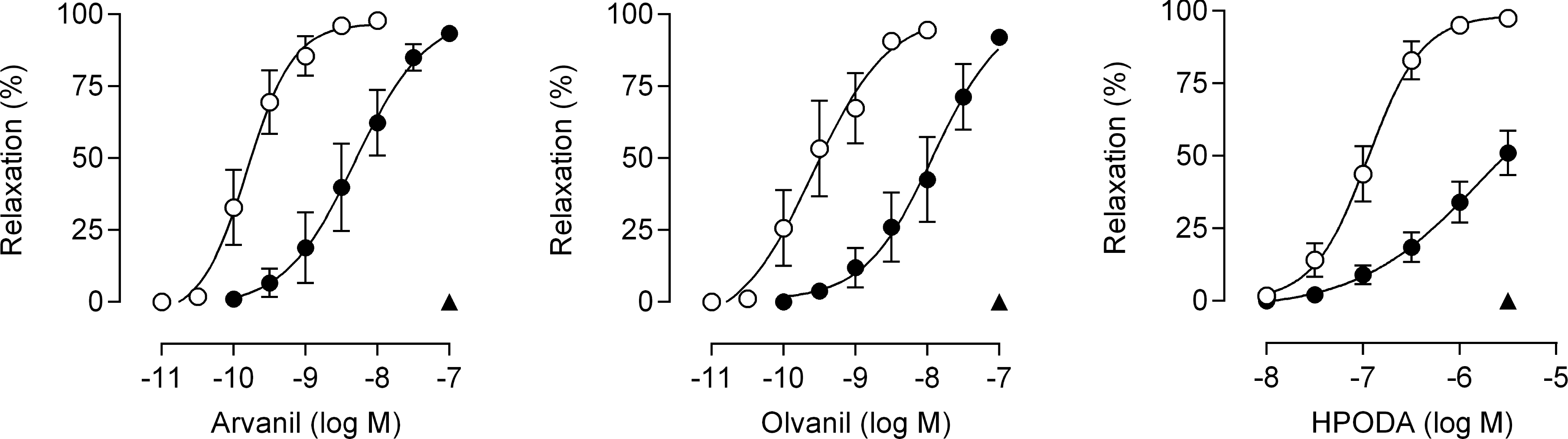

Supplement: Figure S1 — Arvanil, olvanil and N-(4-hydroxyphenyl)-9Z-octadecenamide (HPODA) produce TRPV1-dependent vasorelaxation. The TRPV1 blocker capsazepine significantly suppressed the vasorelaxation evoked by arvanil (p<0.01), olvanil (p<0.01) and HPODA (p<0.001) in rat mesenteric arterial segments precontracted with phenylephrine. Unfilled and filled circles indicate vasodilator responses in the absence (n = 7–12) and presence (n = 6–7) of capsazepine (3 µM), respectively (the former values are the same as in Fig. 1B). Filled triangles indicate responses after pretreatment (60 min) of the vascular segment with capsaicin (1 µM) to inactivate capsaicin-sensitive nerve fibres (n = 6–8). Values are expressed as mean ± SE. Mann-Whitney U test was used to compare the areas under the concentration-response curve in the absence and presence of capsazepine. Sigmoidal concentration-response curves (variable slope) were constructed, using GraphPad Prism 6.0 software (California, USA). (TIF) [file pone.0070690.s001.tif]
